# The genome sequence of the Common Pug, *Eupithecia vulgata* (Haworth, 1809)

**DOI:** 10.12688/wellcomeopenres.19246.1

**Published:** 2023-03-23

**Authors:** Douglas Boyes, John F. Mulley, University of Oxford and Wytham Woods Genome Acquisition Lab, Darwin Tree of Life Barcoding collective, Wellcome Sanger Institute Tree of Life programme, Wellcome Sanger Institute Scientific Operations: DNA Pipelines collective, Tree of Life Core Informatics collective, Darwin Tree of Life Consortium

**Affiliations:** 1 UK Centre for Ecology & Hydrology, Wallingford, England, UK; 2 School of Natural Sciences, Bangor University, Bangor, Wales, UK

**Keywords:** Eupithecia vulgata, Common Pug, genome sequence, chromosomal, Lepidoptera

## Abstract

We present a genome assembly from an individual male *Eupithecia vulgata* (the Common Pug; Arthropoda; Insecta; Lepidoptera; Geometridae). The genome sequence is 454.7 megabases in span. Most of the assembly is scaffolded into 31 chromosomal pseudomolecules, including the assembled Z sex chromosome. The mitochondrial genome has also been assembled and is 17.1 kilobases in length.

## Species taxonomy

Eukaryota; Metazoa; Ecdysozoa; Arthropoda; Hexapoda; Insecta; Pterygota; Neoptera; Endopterygota; Lepidoptera; Glossata; Ditrysia; Geometroidea; Geometridae; Larentiinae; *Eupithecia*; *Eupithecia vulgata* (Haworth, 1809) (NCBI:txid934866).

## Background

The Common Pug is a small (15–18 mm wingspan) Geometrid moth, common across the UK and wider Palearctic, originally named as *Phalaena vulgata* by Adrian Hardy Haworth. Three subspecies are typically recognised in the UK: the widespread *E. vulgata vulgata*; *E. vulgata scotia* from Scotland ( Cockayne, 1951); and *E. vulgata clarensis* from County Clare ( Huggins, 1962), although some authors do not consider the latter two subspecies as valid and propose instead that they should be considered forms ( Riley & Prior, 2003). Common Pugs are readily attracted to light, especially males, and peak flight time in the UK is between mid-May to mid-July, although some individuals have been reported as early as March or as late as September ( NBN Atlas Partnership, 2021), and there can be a second emergence in August, particularly in the south. Larvae are polyphagous, and consume a range of deciduous trees including hawthorn, sallow, and oak, and shrubs and herbaceous plants including bramble, ragworts, hogweed and dandelion. *E. vulgata* was listed as ‘Least concern’ in a recent review of macro-moth status in Great Britain, based on records from 1594 hectads (10 km × 10 km grid squares), far exceeding the ≥15 hectads required to achieve this classification ( Fox *et al.*, 2019).

As with other Pugs, the forewings are held at right angles to the body when at rest, and the hindwings are covered by the forewings. Colouration is variable, with a typically reddish-brown base colour which may or may not include a whitish spot in the trailing corner and a darker discal spot, and usually with pale cross-lines angled at the leading edge. Identification is sometimes complicated by the co-occurrence of several colour morphs, including a melanic form (f. *atropicta* Dietze 1910) and another that lacks cross-lines but maintains the overall ground colour (f. *unicolor* Lempke 1951). As with other melanic moth species, it is possible that the cortex gene underlies the melanic form ( van’t Hof *et al.*, 2019). The genome assembly reported here will aid the testing of this hypothesis and facilitate study of the genetic basis of the widespread colour variation.

## Genome sequence report

**Figure 1.**
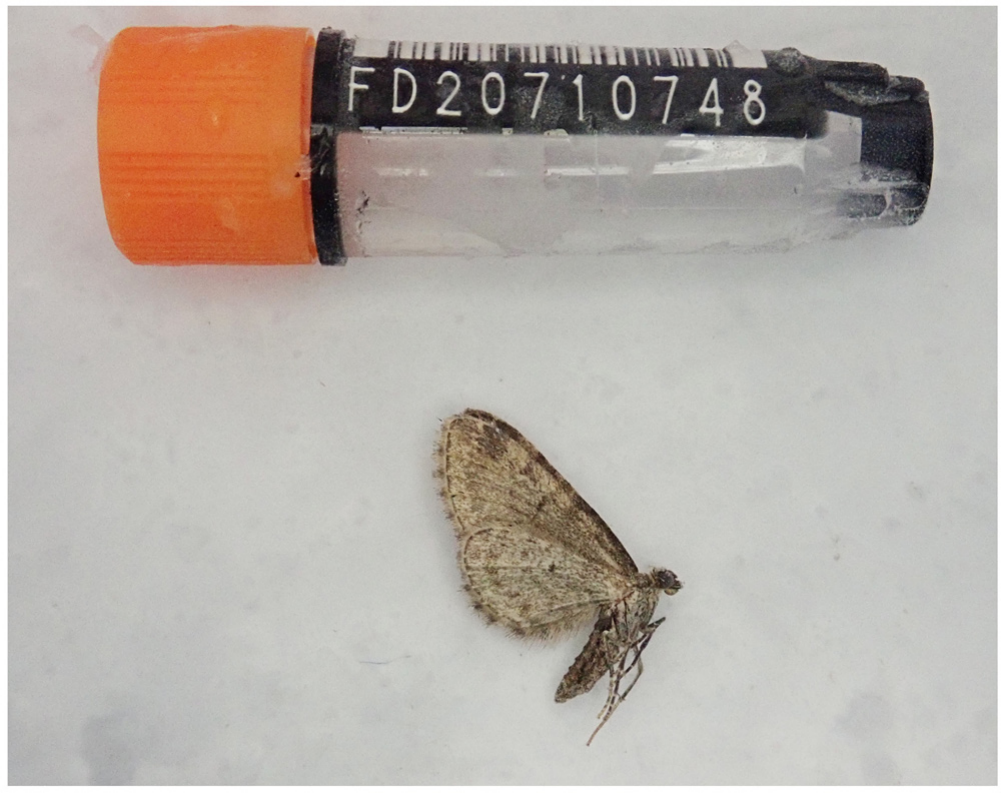
Photograph of the *Eupithecia vulgata* (ilEupVulg1) specimen used for genome sequencing.

The genome was sequenced from one male *Eupithecia vulgata* ( Figure 1) collected from Wytham Woods, Oxfordshire, UK (latitude 51.77, longitude –1.32). A total of 44-fold coverage in Pacific Biosciences single-molecule HiFi long reads was generated. Primary assembly contigs were scaffolded with chromosome conformation Hi-C data. Manual assembly curation corrected nine missing or mis-joins and removed one haplotypic duplication, reducing the scaffold number by 2.44%.

**Figure 2.**
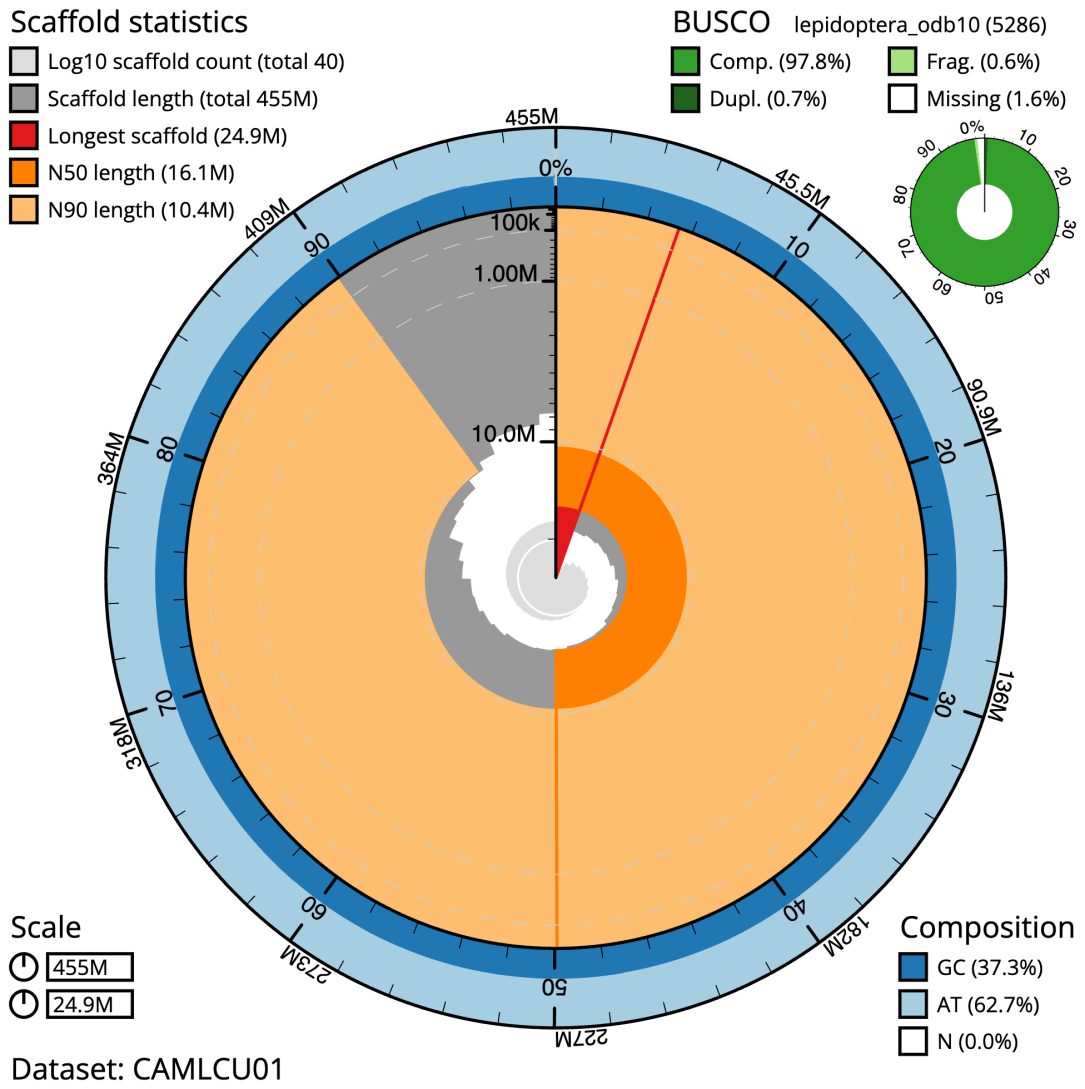
Genome assembly of *Eupithecia vulgata*, ilEupVulg1.1: metrics. The BlobToolKit Snailplot shows N50 metrics and BUSCO gene completeness. The main plot is divided into 1,000 size-ordered bins around the circumference with each bin representing 0.1% of the 454,699,389 bp assembly. The distribution of scaffold lengths is shown in dark grey with the plot radius scaled to the longest scaffold present in the assembly (24,908,255 bp, shown in red). Orange and pale-orange arcs show the N50 and N90 scaffold lengths (16,073,052 and 10,404,322 bp), respectively. The pale grey spiral shows the cumulative scaffold count on a log scale with white scale lines showing successive orders of magnitude. The blue and pale-blue area around the outside of the plot shows the distribution of GC, AT and N percentages in the same bins as the inner plot. A summary of complete, fragmented, duplicated and missing BUSCO genes in the lepidoptera_odb10 set is shown in the top right. An interactive version of this figure is available at https://blobtoolkit.genomehubs.org/view/ilEupVulg1.1/dataset/CAMLCU01/snail.

The final assembly has a total length of 454.7 Mb in 40 sequence scaffolds with a scaffold N50 of 16.1 Mb ( Table 1). Most (99.92%) of the assembly sequence was assigned to 31 chromosomal-level scaffolds, representing 30 autosomes and the Z sex chromosome. Chromosome-scale scaffolds confirmed by the Hi-C data are named in order of size ( Figure 2– Figure 5; Table 2). While not fully phased, the assembly deposited is of one haplotype. Contigs corresponding to the second haplotype have also been deposited. The estimated Quality Value (QV) of the final assembly is 68.5 with *k*-mer completeness of 100%, and the assembly has a BUSCO v5.3.2 ( Manni *et al.*, 2021) completeness of 97.8% (single 97.1%, duplicated 0.7%) using the lepidoptera_odb10 reference set ( *n* = 5,286).

**Table 1.** Genome data for *Eupithecia vulgata*, ilEupVulg1.1.

Project accession data		
Assembly identifier	ilEupVulg1.1	
Species	*Eupithecia vulgata*	
Specimen	ilEupVulg1	
NCBI taxonomy ID	934866	
BioProject	PRJEB54942	
BioSample ID	SAMEA10979141	
Isolate information	ilEupVulg1 (DNA sequencing) ilEupVulg2 (Hi-C scaffolding)	
Assembly metrics *		*Benchmark*
Consensus quality (QV)	68.5	*≥ 50*
*k*-mer completeness	100%	*≥ 95%*
BUSCO **	C:97.8%[S:97.1%,D:0.7%], F:0.6%,M:1.6%,n:5,286	*C ≥ 95%*
Percentage of assembly mapped to chromosomes	99.92%	*≥ 95%*
Sex chromosomes	Z chromosome	*localised homologous pairs*
Organelles	Mitochondrial genome assembled	*complete single alleles*
Raw data accessions		
PacificBiosciences SEQUEL II	ERR10008898	
Hi-C Illumina	ERR9988141	
Genome assembly		
Assembly accession	GCA_946478455.1	
*Accession of alternate haplotype*	GCA_946478135.1	
Span (Mb)	454.7	
Number of contigs	52	
Contig N50 length (Mb)	16.0	
Number of scaffolds	40	
Scaffold N50 length (Mb)	16.1	
Longest scaffold (Mb)	24.9	

* Assembly metric benchmarks are adapted from column VGP-2020 of “Table 1: Proposed standards and metrics for defining genome assembly quality” from ( Rhie *et al.*, 2021). ** BUSCO scores based on the lepidoptera_odb10 BUSCO set using v5.3.2. C = complete [S = single copy, D = duplicated], F = fragmented, M = missing, n = number of orthologues in comparison. A full set of BUSCO scores is available at https://blobtoolkit.genomehubs.org/view/ilEupVulg1.1/dataset/CAMLCU01/busco.

**Figure 3.**
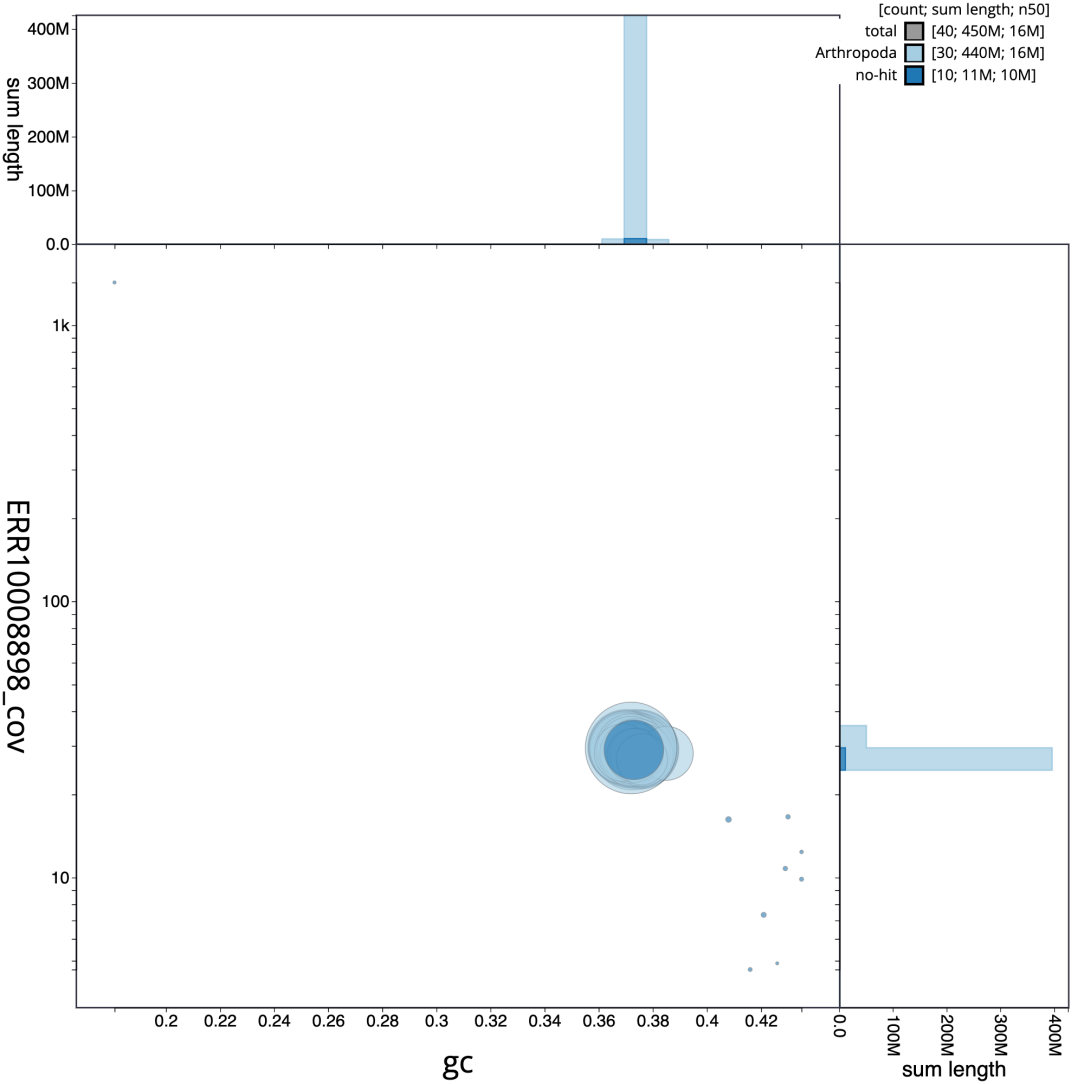
Genome assembly of *Eupithecia vulgata*, ilEupVulg1.1: GC coverage. BlobToolKit GC-coverage plot. Scaffolds are coloured by phylum. Circles are sized in proportion to scaffold length. Histograms show the distribution of scaffold length sum along each axis. An interactive version of this figure is available at https://blobtoolkit.genomehubs.org/view/ilEupVulg1.1/dataset/CAMLCU01/blob.

**Figure 4.**
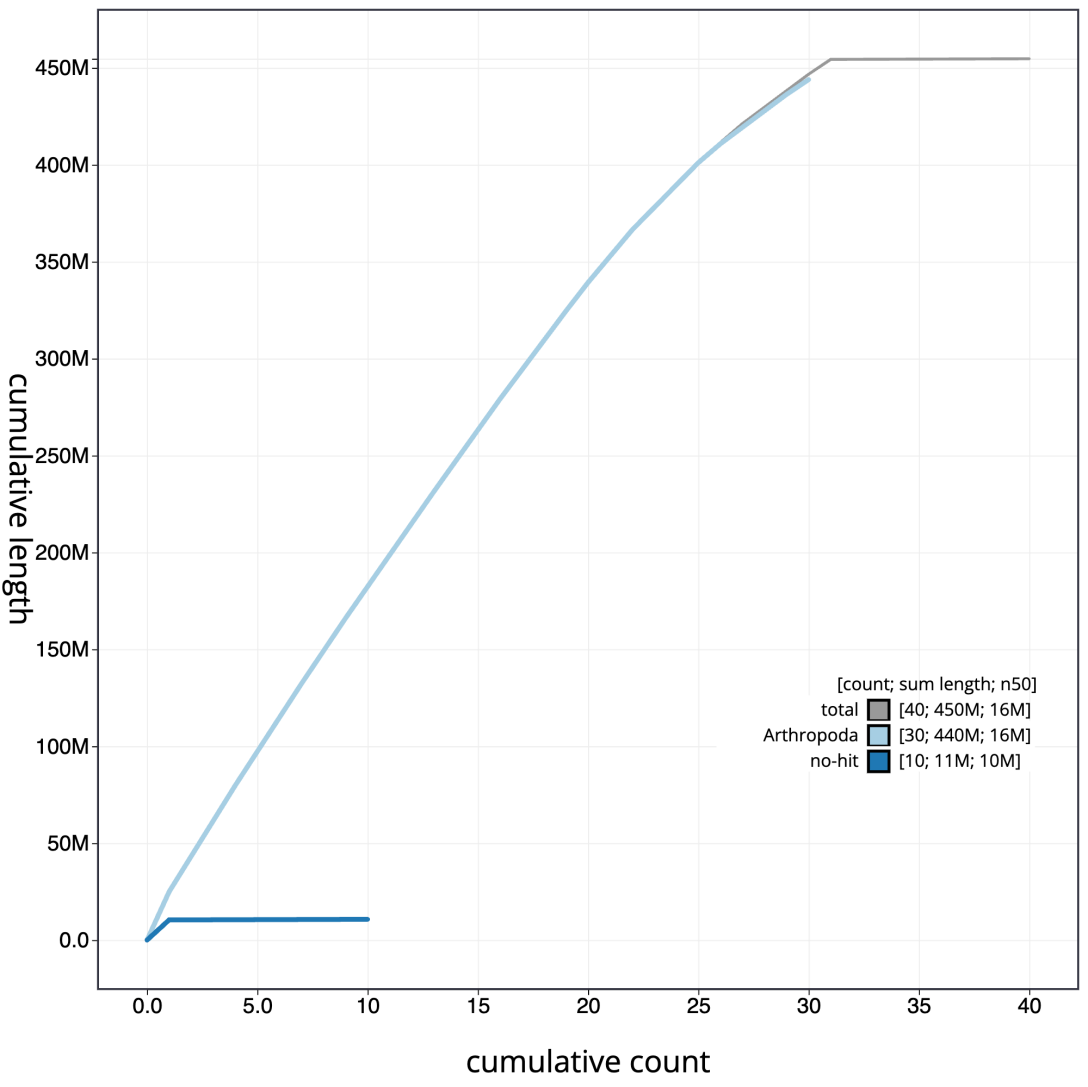
Genome assembly of *Eupithecia vulgata*, ilEupVulg1.1: cumulative sequence. BlobToolKit cumulative sequence plot. The grey line shows cumulative length for all scaffolds. Coloured lines show cumulative lengths of scaffolds assigned to each phylum using the buscogenes taxrule. An interactive version of this figure is available at https://blobtoolkit.genomehubs.org/view/ilEupVulg1.1/dataset/CAMLCU01/cumulative.

**Figure 5.**
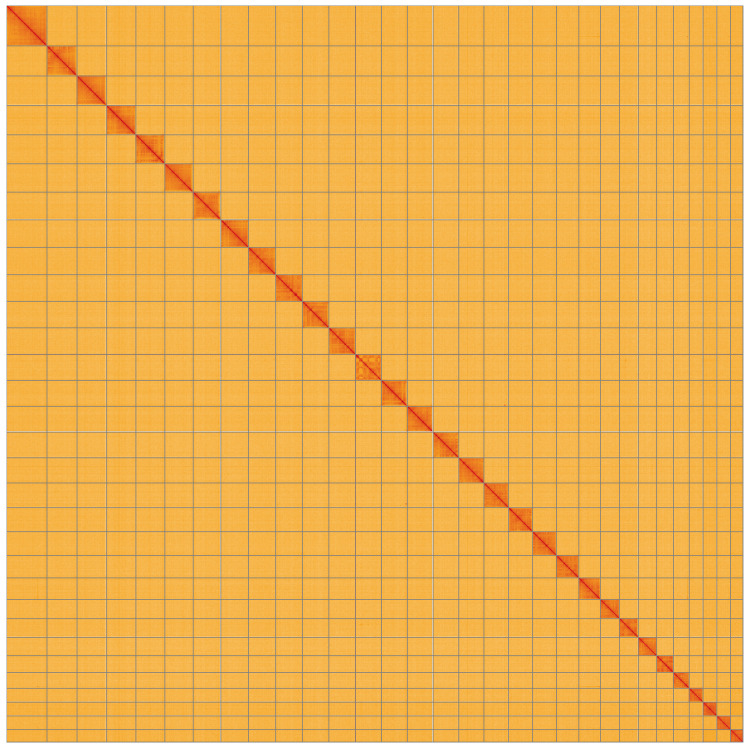
Genome assembly of *Eupithecia vulgata*, ilEupVulg1.1: Hi-C contact map. Hi-C contact map of the ilEupVulg1.1 assembly, visualised using HiGlass. Chromosomes are shown in order of size from left to right and top to bottom. An interactive version of this figure may be viewed at https://genome-note-higlass.tol.sanger.ac.uk/l/?d=K1u-LjpZRhitcCllQNqeCA.

**Table 2.** Chromosomal pseudomolecules in the genome assembly of *Eupithecia vulgata*, ilEupVulg1.

INSDC accession	Chromosome	Size (Mb)	GC%
OX297860.1	1	18.56	37.5
OX297861.1	2	18.28	37.3
OX297862.1	3	18.04	37.5
OX297863.1	4	17.88	37.5
OX297864.1	5	17.46	37.2
OX297865.1	6	17.11	37.2
OX297866.1	7	16.99	37
OX297867.1	8	16.83	37.3
OX297868.1	9	16.44	37
OX297869.1	10	16.38	37.1
OX297870.1	11	16.34	37
OX297871.1	12	16.07	37.5
OX297872.1	13	16	37.3
OX297873.1	14	15.9	37.2
OX297874.1	15	15.87	37
OX297875.1	16	15.49	37.3
OX297876.1	17	15.24	37
OX297877.1	18	14.83	37.5
OX297878.1	19	14.69	37.2
OX297879.1	20	13.81	37.2
OX297880.1	21	13.31	37.4
OX297881.1	22	11.86	37.2
OX297882.1	23	11.54	37.5
OX297883.1	24	11.3	37.2
OX297884.1	25	10.4	37.3
OX297885.1	26	9.72	36.9
OX297886.1	27	8.53	37.4
OX297887.1	28	8.51	38.5
OX297888.1	29	8.46	37.3
OX297889.1	30	7.64	37.6
OX297859.1	Z	24.91	37.2
OX297890.1	MT	0.02	19

## Methods

### Sample acquisition and nucleic acid extraction

Two *Eupithecia vulgata* specimens (ilEupVulg1 and ilEupVulg2) were collected from Wytham Woods, Oxfordshire (biological vice-county: Berkshire), UK (latitude 51.77, longitude –1.32) on 28 May 2021 and 16 June 2021 respectively. The specimens were taken from woodland habitat by Douglas Boyes (University of Oxford) using a light trap. The specimens were identified by the collector and snap-frozen on dry ice.

DNA was extracted at the Tree of Life laboratory, Wellcome Sanger Institute (WSI). The ilEupVulg1 sample was weighed and dissected on dry ice. Whole organism tissue was disrupted using a Nippi Powermasher fitted with a BioMasher pestle. High molecular weight (HMW) DNA was extracted using the Qiagen MagAttract HMW DNA extraction kit. HMW DNA was sheared into an average fragment size of 12–20 kb in a Megaruptor 3 system with speed setting 30. Sheared DNA was purified by solid-phase reversible immobilisation using AMPure PB beads with a 1.8X ratio of beads to sample to remove the shorter fragments and concentrate the DNA sample. The concentration of the sheared and purified DNA was assessed using a Nanodrop spectrophotometer and Qubit Fluorometer and Qubit dsDNA High Sensitivity Assay kit. Fragment size distribution was evaluated by running the sample on the FemtoPulse system.

### Sequencing

Pacific Biosciences HiFi circular consensus DNA sequencing libraries were constructed according to the manufacturers’ instructions. DNA sequencing was performed by the Scientific Operations core at the WSI on Pacific Biosciences SEQUEL II (HiFi) instrument. Hi-C data were also generated from whole organism tissue of ilEupVulg2 using the Arima v2 kit and sequenced on the Illumina NovaSeq 6000 instrument.

### Genome assembly, curation and evaluation

Assembly was carried out with Hifiasm ( Cheng *et al.*, 2021) and haplotypic duplication was identified and removed with purge_dups ( Guan *et al.*, 2020). The assembly was scaffolded with Hi-C data ( Rao *et al.*, 2014) using YaHS ( Zhou *et al.*, 2023). The assembly was checked for contamination as described previously ( Howe *et al.*, 2021). Manual curation was performed using HiGlass ( Kerpedjiev *et al.*, 2018) and Pretext ( Harry, 2022). The mitochondrial genome was assembled using MitoHiFi ( Uliano-Silva *et al.*, 2022), which performed annotation using MitoFinder ( Allio *et al.*, 2020). To evaluate the assembly, MerquryFK was used to estimate consensus quality (QV) scores and *k*-mer completeness ( Rhie *et al.*, 2020). The genome was analysed and BUSCO scores ( Manni *et al.*, 2021; Simão *et al.*, 2015) were generated within the BlobToolKit environment ( Challis *et al.*, 2020). Table 3 contains a list of software tool versions and sources.

**Table 3.** Software tools: versions and sources.

Software tool	Version	Source
BlobToolKit	4.0.7	https://github.com/blobtoolkit/blobtoolkit
BUSCO	5.3.2	https://gitlab.com/ezlab/busco
Hifiasm	0.16.1-r375	https://github.com/chhylp123/hifiasm
HiGlass	1.11.6	https://github.com/higlass/higlass
Merqury	MerquryFK	https://github.com/thegenemyers/MERQURY.FK
MitoHiFi	2	https://github.com/marcelauliano/MitoHiFi
PretextView	0.2	https://github.com/wtsi-hpag/PretextView
purge_dups	1.2.3	https://github.com/dfguan/purge_dups
YaHS	yahs-1.1.91eebc2	https://github.com/c-zhou/yahs

### Ethics and compliance issues

The materials that have contributed to this genome note have been supplied by a Darwin Tree of Life Partner. The submission of materials by a Darwin Tree of Life Partner is subject to the Darwin Tree of Life Project Sampling Code of Practice. By agreeing with and signing up to the Sampling Code of Practice, the Darwin Tree of Life Partner agrees they will meet the legal and ethical requirements and standards set out within this document in respect of all samples acquired for, and supplied to, the Darwin Tree of Life Project. All efforts are undertaken to minimise the suffering of animals used for sequencing. Each transfer of samples is further undertaken according to a Research Collaboration Agreement or Material Transfer Agreement entered into by the Darwin Tree of Life Partner, Genome Research Limited (operating as the Wellcome Sanger Institute), and in some circumstances other Darwin Tree of Life collaborators.

## Data Availability

European Nucleotide Archive: *Eupithecia vulgata* (common pug). Accession number PRJEB54942; https://identifiers.org/ena.embl/PRJEB54942. ( Wellcome Sanger Institute, 2022) The genome sequence is released openly for reuse. The *Eupithecia vulgata* genome sequencing initiative is part of the Darwin Tree of Life (DToL) project. All raw sequence data and the assembly have been deposited in INSDC databases. The genome will be annotated using available RNA-Seq data and presented through the Ensembl pipeline at the European Bioinformatics Institute. Raw data and assembly accession identifiers are reported in Table 1.

## Author information

Members of the University of Oxford and Wytham Woods Genome Acquisition Lab are listed here: https://doi.org/10.5281/zenodo.4789928.

Members of the Darwin Tree of Life Barcoding collective are listed here: https://doi.org/10.5281/zenodo.4893703.

Members of the Wellcome Sanger Institute Tree of Life programme are listed here: https://doi.org/10.5281/zenodo.4783585.

Members of Wellcome Sanger Institute Scientific Operations: DNA Pipelines collective are listed here: https://doi.org/10.5281/zenodo.4790455.

Members of the Tree of Life Core Informatics collective are listed here: https://doi.org/10.5281/zenodo.5013541.

Members of the Darwin Tree of Life Consortium are listed here: https://doi.org/10.5281/zenodo.4783558.
