# Supplementary material for: The genome sequence of the Common Pug, Eupithecia vulgata (Haworth, 1809)
Source: Wellcome Open Res. 2023 Mar 23;8:129. [Version 1] doi: 10.12688/wellcomeopenres.19246.1 (PMC10808854; doi:10.12688/wellcomeopenres.19246.1)
Supplement: Peer review history

# Reviewer report: Reviewer response for version 1

**DOI:** 10.21956/wellcomeopenres.21329.r72067

**Published:** 2024-01-26

**Authors:** Satoshi Yamamoto

**Affiliations:** 1 Graduate School of Science, Kyoto University, Kyoto, Japan

This paper reports on a De novo genome assembly of *Eupithecia vulgata*. In this study, Pac Bio Hifi reads were obtained from a single male individual. Hi-C data was also obtained from another individual to do scaffolding, and as a result, chromosome-scale genome sequences were reported.

 The description of methods is reproducible enough. The whole genome sequence and Hi-C data are also publicly available.

Are sufficient details of methods and materials provided to allow replication by others?

Yes

Is the rationale for creating the dataset(s) clearly described?

Yes

Are the datasets clearly presented in a useable and accessible format?

Yes

Are the protocols appropriate and is the work technically sound?

Yes

Reviewer Expertise:

Molecular ecology

I confirm that I have read this submission and believe that I have an appropriate level of expertise to confirm that it is of an acceptable scientific standard.

# Reviewer report: Reviewer response for version 1

**DOI:** 10.21956/wellcomeopenres.21329.r72056

**Published:** 2024-01-26

**Authors:** Fabrice Legeai

**Affiliations:** 1 INRAE, UMR, Institute of Genetics, Environment and Plant Protection, University of Rennes, Rennes, France

This paper presents the genome assembly of the common pug, Eupithecia vulga. The quality of the raw data is excellent and the sequencing and assembly methods employed are appropriate. Consequently, the resulting assembly reachs a high level of quality.

 However, I have a few minor concerns regarding the manuscript that require further details for a more comprehensive evaluation:

- It would be beneficial to include information about existing genomics resources for the Eupitheciae genus. For instance, acknowledging that the genome of Eupithecia dodoneata has already been sequenced by DToL would enhance the context.
- Although 31 chromosomes were recovered, it remains unclear if this corresponds with the expected number determined by alternative techniques or in comparison to close species.
- Providing summary statistics for the generated raw HiFi data, such as the number of reads, N50, mean, and median size, would assist in assessing the raw data's quality.
- The depth of coverage for Hi-C data should be explicitly mentioned.
- Clarification is needed on how k-mer completeness was calculated, and provide corresponding plots or statistics.  Also an estimation of heterozygosity level would be informative and should be included.
- Specify the methodology employed to select the Z chromosome from the assembly. If based on read coverage, include this data in Table 2.
- To verify if the chromosomes reach the telomere, search for telomere motifs at the ends of the scaffolds.
- Explain why the mitochondrial genome was not included in the primary assembly, and needed to be assembled from reads apart with MitoHiFi.
- Scrutinize the scaffolds with higher GC content and lower coverage, labeled as arthropod in the blobtoolkit Figure 3, and conduct, if possible, more investigation in order to precise their origin (for instance colinearity with other pugs genome).
- For reproductibility, please provide the parameters used for each software run, if any ; otherwise write  'default parameters'.

Lastly, consider complementing the genome with annotation data, including RNASeq, and conducting comparisons with other pug genomes, such as the oak-tree pug. This additional information would enrich the manuscript.

Are sufficient details of methods and materials provided to allow replication by others?

Partly

Is the rationale for creating the dataset(s) clearly described?

Yes

Are the datasets clearly presented in a useable and accessible format?

Yes

Are the protocols appropriate and is the work technically sound?

Yes

Reviewer Expertise:

bionformatics, insect genomics.

I confirm that I have read this submission and believe that I have an appropriate level of expertise to confirm that it is of an acceptable scientific standard, however I have significant reservations, as outlined above.

# Reviewer report: Reviewer response for version 1

**DOI:** 10.21956/wellcomeopenres.21329.r72068

**Published:** 2024-01-24

**Authors:** Markus Friedrich

**Affiliations:** 1 Department of Biological Sciences, Wayne State University, Detroit, Michigan, USA

**This a concise report of the sequencing of the genome  of the Common Pug, Eupithecia vulgata, in the context of the Wellcome Sanger Institute Tree of Life programme.**

**Only one comment:**

**“The ilEupVulg1 sample was weighed and dissected on dry ice.”**

**Which parts were removed and subjected to DNA extraction?**

Are sufficient details of methods and materials provided to allow replication by others?

Yes

Is the rationale for creating the dataset(s) clearly described?

Yes

Are the datasets clearly presented in a useable and accessible format?

Yes

Are the protocols appropriate and is the work technically sound?

Yes

Reviewer Expertise:

Comparative genomics of arthropods

I confirm that I have read this submission and believe that I have an appropriate level of expertise to confirm that it is of an acceptable scientific standard.

# Reviewer report: Reviewer response for version 1

**DOI:** 10.21956/wellcomeopenres.21329.r70597

**Published:** 2024-01-02

**Authors:** Marco Gerdol

**Affiliations:** 1 University of Trieste, Trieste, Italy

The manuscript by Boyes and colleagues reports the genome sequence of the Common Pug Eupithecia vulgate, which is part of the Darwing Tree of Life project. As such, the manuscript reports a genome assembly of high quality, obtained with a highly standardized and reproducible pipeline. The methodologies are clear and reproducible and the report of assembly metrics is straightforward and easy to follow. I only have a very few minor comments that the authors may choose to address at their discretion.

 “31 chromosomal-level scaffolds, representing 30 autosomes and the Z sex chromosome” -> was this in line with previous cytogenetic estimates (if available?)

 As a general comment I usually always make to any genome paper, having a rough k-mer based estimate of heterozygosity would be useful for anybody interested in planning future population genomics studies on this species.

Are sufficient details of methods and materials provided to allow replication by others?

Yes

Is the rationale for creating the dataset(s) clearly described?

Yes

Are the datasets clearly presented in a useable and accessible format?

Yes

Are the protocols appropriate and is the work technically sound?

Yes

Reviewer Expertise:

invertebrate genomics

I confirm that I have read this submission and believe that I have an appropriate level of expertise to confirm that it is of an acceptable scientific standard.
